# The Life Process of Children Who Survived the Manjil Earthquake: A Decaying or Renewing Process

**DOI:** 10.1371/currents.dis.dd88534c0ab58b02d225709b77c861a0

**Published:** 2017-04-04

**Authors:** Abbas Shamsalinia, Fatemeh Ghaffari, Nahid Dehghan–Nayeri, Sarieh Poortaghi

**Affiliations:** School of Nursing and Midwifery, Ramsar Nursing Care Research Center, Babol University of Medical Sciences, Mazandaran, Iran.; Assistant Professor in Nursing, Babol University of Medical Sciences, Mazandaran, Iran; School of Nursing and Midwifery, Nursing and Midwifery Care Research Center, Tehran University of Medical Sciences, Tehran, Iran; Department of Community Health Nursing, School of Nursing and Midwifery, Tehran University of Medical Sciences, Tehran, Iran

**Keywords:** Earthquakes, Grounded theory, Natural disaster, Survivors

## Abstract

**Introduction:**

Among earthquake survivors, children are more vulnerable than other age groups due to their exposure to harrowing scenes of devastation as well as their drastically new living situations that result from an earthquake disaster. The life process of children survivors undergoes many different changes that are affected by a wide range of factors. Understanding the life process of these children may lead to effective outcomes and interventions. In addition, observing children survivors establishes knowledge and understanding of the challenges that correspond with earthquake disasters. Further, observing this group may be further effective in decision-making and establishing types of assistance in similar circumstances.

**Objectives:**

This study was done to explain the life process of children who survived the earthquake of Manjil in northern side of Iran.

**Methods:** This qualitative study is based on the grounded theory approach. The sampling involved purposive interviews with 12 children who survived the Manjil earthquake and were under 12 years of age at the time of the earthquake. The initial interviews were followed by continuous comparative analysis, and thus the sampling process adopted a theoretical trend. In the end, by the formation of categories and the central variable of the study, interviews were conducted with 16 subjects and sufficient data was provided. Data was collected through face-to-face, in-depth interviews using an interview guide. In order to enrich the categories formed in data analysis, we had also 6 telephone interviews with the same participants in order to complete missed needed information. Data collection began in 2015 and continued up until 2016. Data was analysed using the Strauss-Corbin approach.

**Results:**

The life process of children earthquake survivors consists of ‘unexpected encounter’, ‘transient relief activities’ and ‘long-lasting consequences’. The central variable of this study is ‘the dark shadow of pain and the light shadow of life expectancy’. The life experience of this group of children is immersed in painful memories and varies under different conditions.

**Discussion and Conclusion:**

According to the results of this study, one of the factors affecting the lives of children earthquake survivors which could threaten their health is providing non-specific and transient services. Training relief staff to consider the specific needs of these children at the time of the rescue operation could contribute to improving their health level in various aspects. Considering the effective and comprehensive rehabilitation program in Disaster Management by policymakers can prevent permanent complications caused by earthquakes. Planning and taking action to identify misbehaviours in this group of children as well as raising public awareness, particularly for parents, on how to manage the outcomes of natural disasters are some of the most significant public health priorities. Providing public mental health services for parents and children who survive an earthquake helps to address potential psychological problems in this group of survivors.

## Background

The Manjil earthquake^1^ occurred on June 21, 1990 at 21 GMT near the town of Rudbar, in villages of Gilan Province and the northwest region of Zanjan Province, Tarom Olya located in the north-west of Iran. The Manjil earthquake caused great human and financial losses within a 100-kilometer radius of the epicentre. The earthquake occurred in Rudbar and Manjil was one of the largest and most destructive earthquakes in Iran in recent decades. The earthquake caused nearly 35,000 deaths and 60,000 injuries, and left more than 500,000 people homeless. In addition, 200,000 residential units were demolished, 60,000 of which were completely destroyed. Due to the landslides that occurred during the Manjil earthquake, villages were deeply buried. The initial damage caused by the Manjil earthquake was estimated to amount to more than 800 billion rials, and the disaster caused economic losses equal to 2.5 percent of the gross national product^2^.

## Introduction

Published statistics of disasters around the world show that in the past two decades, more than 4.3 million people have died, millions of people have been injured and tens of billions of dollars have been spent on financial and life compensations due to natural disasters^3^. Due to the climatic and geographical situation of Iran, it is one the high-risk countries in terms of natural disasters^4^. Advances in emergency and injury care systems have caused survivors of natural disasters need wide physical, psychological and social recoveries^5^.

Among the survivors of the earthquakes, children are more vulnerable than other age groups due to the fact that they are exposed to distressing scenes of devastation. In addition, children survivors are especially vulnerable as their living situations immensely shift following an earthquake disaster. If the special needs of these victims do not receive urgent attention, the evolutionary process of their growth will undoubtedly be interrupted, and they will face serious physical and psychological effects in the near or distant future. Most planning programs emphasise the most immediate needs of disaster victims, including rescue and relief activities and primary care^6^.

However, it should be noted that in addition to the significant effects and damages associated with natural disasters, the impact of such disasters on victims’ quality of life is much deeper and severe. Therefore, it is crucial to improve the health of victims for prolonged periods after the disaster. Nevertheless, many of the studies conducted in this field have not taken a comprehensive look at this process, and have usually focused on only one aspect such as psychological interventions after disasters, post-disaster stress disorders^7^^,^^8^, physical injuries^9^, and the role of community participation in physical reconstruction^10^. Since few studies have specifically explained the lives of children who have survived natural disasters, this study seeks to investigate this particular group. Further, studying children survivors in this context is necessary due to the particular context and culture of Iranian society as it differs from other countries which is based on Islamic beliefs and traditional-local customs. Understanding the life process of these children may lead to effective outcomes and interventions, and explaining the life process of these children according to their experiences could lead to a better understanding of their needs, challenges, issues and problems following their experiences of natural disasters. Furthermore, such a study may reveal the challenges resulting from earthquakes, and may also provide results that are applicable to similar circumstances. Therefore, to address this gap in research, this study aims to explain the life process of children who have survived earthquakes.

## Methods

This study was conducted using the grounded theory approach, a qualitative research method based on symbolic interactionism. This study examines the social processes in the context of human interactions^11^.

The sampling involved purposive interviews with 12 children who survived the Manjil earthquake and were under 12 years old at the time of the earthquake. The initial interviews were followed by continuous comparative analysis, and the sampling process therefore took a theoretical trend. Based on the formation of categories and the central variable of the study, interviews were conducted with 16 subjects, and sufficient data for ensuring about the data saturation was provided as a result. Data was collected through face-to-face, in-depth interviews using an interview guide In order to enrich the categories formed in data analysis, we had also 6 telephone interviews with the same participants in order to complete missed needed information. The interviews began with general questions and progressively involved more detailed questions regarding participants’ responsiveness. Questions included, ‘Can you tell me about your experiences after the earthquake?’ and ‘How was your life?’ Interviews were performed in a quiet atmosphere according to participants' preferences, in locations such as their homes or in a park. According to the tolerance, willingness and environmental factors of participants, 22 interviews were conducted for this study. The duration of face-to-face interviews was 25 to 45 minutes, and telephone interviews lasted 15 to 20 minutes. Data collection started in 2015 and continued up to 2016.

**Data Analysis**

Data was analysed using the Strauss-Corbin approach^11^. According to this method, the current study respectively employed concept analysis (immediately after the first interview), context analysis, process analysis, and then combined and integrated the categories and proposed the basic theory (writing analytical stories and reviewing the reminders). The audio-recorded interviews were transcribed, and the typed texts were reviewed several times. Then, the texts were studied line-by-line and word-by-word, and a code was assigned to each key word or sentence. Similar codes were then put together in a category, and the primary classification of 598 codes was obtained. Constant comparison was used to determine the relationship between categories and search the underlying process of data. Then, the researcher integrated the categories using various diagrams to identify the central variable.

**Trustworthiness**

The authenticity of the data was examined using the four criteria of Guba and Lincoln^12^. The data collection took nine months using a combination of face-to-face interviews and telephone interviews. Results were confirmed through peer check and member check. Researchers contributed to the transferability of the data through in-depth descriptions and analysis. The sampling technique with maximum variation (the age at the time of the disaster, gender, losing parents in the disaster, occupation, education level and marital status) was also used, and this improved the suitability or transferability of findings. The researcher precisely recorded and reported on the research process to allow others to investigate the article.

**Ethical Consideration**

Permission for the study was obtained from the ethics committee of the University Of Medical Sciences Of Babol. Related departments were informed of the study objectives, and their written consent to participate in the study was obtained. The time and place of the interview was determined with the agreement of participants, who were assured of the confidentiality of all personal information and interviews as well as the anonymity of all documents related to the research. The results of the study were made available to the participants upon request. All moral standards relating to the use and publication of texts was observed. Permission to record participants’ voices was obtained. Only one of the researchers listened to the recordings. The recorded interviews did not include any personal identification information. Participants were informed that they could withdraw their information or remove themselves from the study altogether at any time.

## Results

In this study, 16 individuals (adults at the time of the study) who were child survivors of the Manjil earthquake were interviewed (Table 1).

**Figure:**
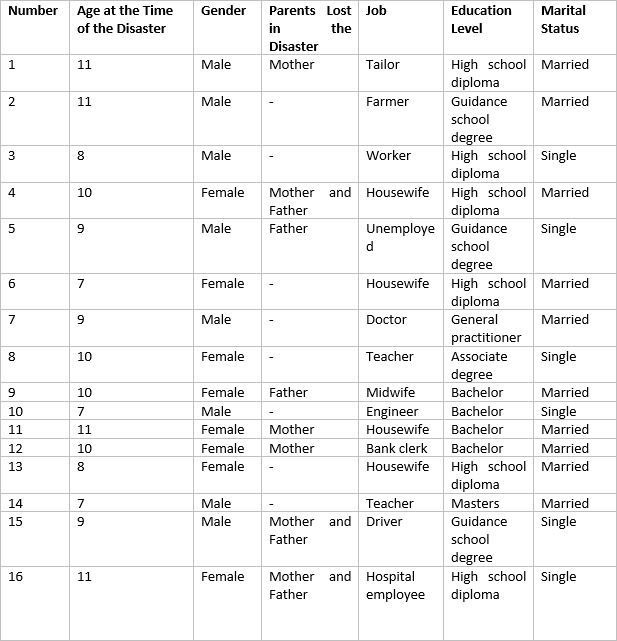
Table 1. Participants' personal information

Analysis of the data resulted in the obtainment of 598 primary codes. During the process of data analysis, six main categories were extracted.

The life process of children survived earthquakes consists of ‘unexpected encounter’, ‘transient relief activities’ and ‘long-lasting consequences’. The central variable of this study is ‘the dark shadow of pain and the light shadow of life expectancy’. This process is influenced by ‘internal factors’ and ‘modifying factors’ (Table 2).

**Figure:**
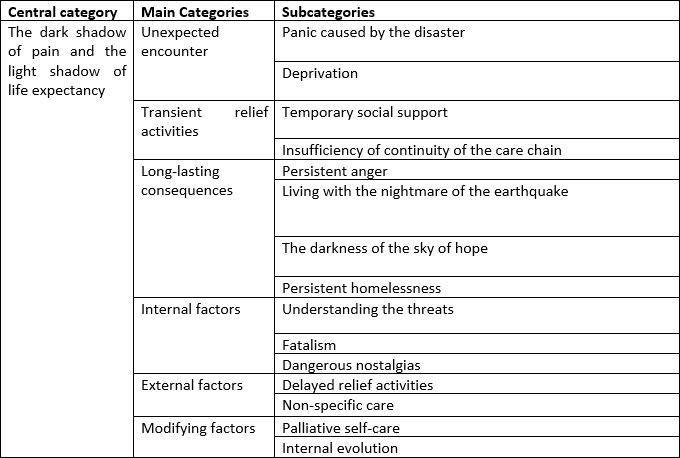
Table 2. Categories extracted from the data

**Unexpected Encounter**

Most of these children were sleeping at the time of the earthquake. Therefore, the fact that they did not anticipate the earthquake or its consequences is associated with reactions of panic and feelings of deprivation.

**Panic Caused by the Disaster**

The first hours after the earthquake were associated with a great deal of fear, apprehension and horror for the majority of these children. These feelings multiplied when they were exposed to the resulting devastation, such as the destruction of shelters and scenes of family members being buried under the rubble, as well as being exposed to wounded and dead victims.

*"The ongoing conditions were like the Resurrection. Destroyed and burnt houses, cracked roads, blood, broken bones, dead bodies along the road etc. Everything was a proof of the Resurrection. I was terrified when my uncle was burying his sister, nieces, nephews and other relatives." (Code 16)*

**Deprivation**

The deep influences of the disaster appeared a few days after the shock and horror of the earthquake. Most children either lost their parents, or their parents were mourning for the loss of their children or other family members. What were previously conceived of as the neighbourhood, kindergarten, school, family and relatives were no longer present. There was no sign of love or loving atmosphere in the family. Most family members were looking for a piece of bread to eat and shelter to live in, and were struggling with many mental pressures. Sometimes the needs of these children were forgotten due to the disastrous situation. Most of these children had no place to sleep and were looking for a clue of memories of good old days in the ruins. Those who had lost their parents and family members wandered around the ruins and cried, and were in search of a safe haven in a world of fear and helplessness. These children were tired, hungry and looking for a way out.

*"The earthquake is nothing but the death of love. All my relatives were buried in front of my eyes, the sky of hope was dark and the pain of this separation grew bigger, and as I grew up, it grew up, too." (Code 14)*

Loneliness and the lack of social security in the initial chaos after the earthquake exposed children survivors to mistreatment. This group of survivors trusted people who were available in the tense and cluttered atmosphere, and exposed themselves to mistreatment. On the other hand, these children were neglected or mistreated due to the preoccupation of police, aid workers and family members who were involved in removing corpses and victims from under the rubble. In addition, some children faced threats such as robbery or getting lost in the process of being delivered to the authorities.

*"I was crying in a street among the ruins in that confusion, and I saw a man coming towards me. He told me that he came to take me to my mother. As soon as he saw nobody was noticing, he took my bangle by force in such a way that my hand was wounded, he put his hand on my mouth so that I wouldn't be able to shout and he threatened me." (Code 13)*

**Transient Relief Activities**

After some time and immediate support efforts, relief activities were provided to enable reconstruction and rehabilitation. However, these steps involved rebuilding houses and buildings, while the important principle of reviving the lives of these children faded out. This category contains the two subcategories of ‘temporary social support’ and ‘ insufficiency of continuity of the care chain’.

**Temporary Social Support**

What appears in the early hours following a disaster is the influx of government and relief aids to disaster-stricken areas. However, after a short period of time, emotions subside and the amount of aid decreases. This especially diminishes significantly as time goes on. In fact, disaster activities are only vaguely recalled.

*"There were lots of people and aids during the first days. But very soon, after one month everything was over. It was [as though] as if the earthquake had involved people only for those days." (Code 15)*

The concept of ‘forgotten’ refers to temporary social support. Although these children require compassionate care, most of them receive humanitarian support for just a few months following a disaster and are gradually deprived of loving attention.

*"Children who have survived earthquakes are highly vulnerable and can be emotionally injured by the smallest issues. They need love and a safe shelter more than anything else." (Code 12)*

**Insufficiency of Continuity of the Care Chain**

A few months after a disaster occurs, continuous care is halted and survivors face serious physical and mental consequences related to the event. The majority of physical and mental health care is limited to emergency and acute care, and rehabilitation services are omitted. The majority of children deal with several complications due to a lack of long-term care services management and planning. ‘**Discontinuous follow-ups**’ have caused most survivors to suffer from multiple chronic physical and mental problems.

*"I was wounded when the earthquake occurred. My leg was stuck under the ruins of the house and was injured. They took me to the hospital and I was taken care of there, but after that they took me home. My parents couldn't afford to spend money for me. Unfortunately, I limp. If somebody cared about us then and thought [about] what would happen next, I wouldn't be like this." (Code 18)*

**Long-lasting Consequences**

Consequences of earthquakes are often delayed and persistent. This category consists of subcategories including ‘persistent anger’, ‘living in the nightmare of the earthquake’, ‘darkness of the sky of hope’ and ‘persistent homelessness’. The consequences of the earthquake proved to be of lower intensity for children who did not lose their parents in the earthquake and could benefit from their physical and mental protection.

**Persistent anger**

After the earthquake, the games and carelessness of childhood shift toward adult behaviours. ‘The repetitive scenario of the earthquake’ replaces game-playing and living a happy life. Teenagers also experience a shift in perception as they too experience undesirable physical, economic and social conditions. The majority of children survivors cannot enjoy their childhood years as their welfare, physical condition and psychological capacity are lacking in the wake of a disaster. Therefore, this group of survivors refers to their adolescence as ‘**unloaded clusters**’ The continuous use of wording such as ‘**sorrowful dialogues**’ or ‘**tragedies**’ describe the significance of sad memories of the past experienced in their minds and souls. Their spirit is obscured by these dialogues, and these survivors live with the daily repetition of parts of their painful past and memories of the earthquake. Further, these survivors attempt to pay homage to their family members, classmates and friends by keeping their memorabilia. In other words, they consider forgetting the event as a source of guilt and disloyalty.

*"At the time I should have been supported by my family, I was taken to an orphanage. As I grew up, I had to leave my friends and move to another place. These changes made me suffer. I didn't understand how my teenage years passed." (Code 4)*

**Living in the Nightmare of the Earthquake**

One of the long-lasting and negative consequences for this group is living in the nightmare of the earthquake. The fear of the earthquake’s recurrence along with continuous insecurity and anxiety are always present. Most of these survivors are deprived of a normal life. Participants expressed that they live in fear not just of the earthquake’s recurrence, but also of other disasters occurring. This fear leads them to experience anxiety. Living with prolonged and continuous anxiety leads to conditions such as depression, psychiatric disorders such as drug abuse, and behavioural problems such as delinquency. Living in the nightmare of the earthquake causes ‘excessive sensitivity’ to others, especially family members. Such behaviours of risk aversion cause these people to draw on their memories of the earthquake and, as a result, deter family members from what they perceive to be possible hazards. This problem is associated with fear, and limits the social lives of victims and their family members. In turn, this brings about dysfunction in the relationships between victims and their family members.

*"I've grown up and aged, but you know, I start shaking even when it's windy. I go and sit in a corner of the room, hug my baby and get terrified. That fear is still with me." (Code 11)*

**The Feeling of Hopelessness**

Some of these survivors believe that the feelings of safety and health have disappeared in their lives and have been substituted by fear, uncertainty and loneliness. They reported that after the earthquake, they feel they have lost their direction in life and have lost all hope. They experience a life without financial and emotional support. Their lives have undergone many major changes, and the grief of losing family members and experience of permanent fear have replaced their normal routine of life. Those who have medical problems and severe physical complications caused by the earthquake are exposed to constant medical procedures and treatment recommendations.

*"I was young at that time. Our financial situation wasn't so bad. But suddenly, we lost everything. My father died hopelessly after a few years. I had to work in a factory. It was very hard. I had to work instead of study." (Code 13)*

The ‘**guilt**’ the respondents feel as the sole survivor, as well as the guilt associated with burying their loved ones, is a significant factor contributing to their feelings of despair. Most of them saw their family members stuck under the rubble and reported that they feel guilty because they could not rescue them.

*"I suffered for a long time. I wished it wasn't me but my parents who were alive. I was little and injured then. I was confused and frightened, and I couldn't do anything for my family. It hurts me a lot." (Code 4)*

For this group of survivors, disappointment leads to a lack of desire and effort in social activities. Thus, they always feel that they are a ‘**social burden**’. Lack of adequate physical and academic qualifications for acquiring a job affects this process. Most of these individuals are not married and live with their relatives because they have lost their families. This problem enables the formation of the feeling of being a social burden.

*"I went my uncle’s house in another city. That poor man had some children and didn't have a good financial situation. He was a farmer. I was a burden to their family. I sometimes realised that they couldn't help doing favours for me, but I also understood that they couldn't afford to provide for their own children." (Code 16)*

Disappointment causes this group of survivors to refer to their current life situation as ‘bubbles on the water’ (they always think that everything is temporary and is not going to last long like a bubble), and keeps them from envisioning a future for themselves. They feel abandoned because their identity has been buried under the rubble of grief and misery. The missing future is represented in impairment, inability to support family expenses, lack of financial resources for marriage, losing parents and their emotional and financial support. In fact, these individuals believe that they feel they are not in control of their destiny or have failed to fulfill their destinies. The fear of a life without financial resources and family support is always with them.

*"A long time ago, I realised that I lost everything in the earthquake. I have neither a good job nor money. I try more and more, but I am always on the first step." (Code 2)*

**Persistent Homelessness**

A few days after the earthquake, some children who lost their parents and did not have anybody to claim them were taken to children care centres. In their opinion, wandering in these centres and waiting to find a safe haven was the worst experience of their lives. They described the separation from family members, relatives, friends and their hometown in a very bitter manner. Some survivors were given to new families and thus faced many new problems. Orphans who were sent to welfare centres or new families grew up in educational systems that differed from their native culture. Sometimes, this way of life proved contrary to these survivors’ previous beliefs and life experiences, and thus led to behavioural and mood disorders. According to survivors, sympathy was the major factor in their acceptance to these new families or centres. However, over time, many children survivors once again experienced homelessness due to behavioural disorders and adversities.

On the other hand, the lack of necessary physical and mental conditions for adoption led some of these children to stay in care centres for a long time. Further, such conditions at times caused them to wander between different centres as they grew older or their physical or mental complications increased. Children without guardians were taken to welfare centres. There was also a significant difference between genders in child adoption such that most boys were adopted in a short time while girls spent a long time on the waiting list.

*"I stayed in the nursery for a long time. Those who were with me were adopted by their relatives or new families. I was sick, I think that's the reason." (Code 15)*

An important point revealed through data analysis was that some of the parents of children survivors were noted as ‘risky parents’. These parents pose risk factors in the physical, mental, moral and social development of their children due to the loss of their own parents or other children, or due to multiple physical injuries caused by the earthquake. These people cannot provide a safe haven for their children to live. Escape, isolation, and anxiety are just some of the outcomes and emotional experiences of children living with risky parents.

*"At first, my father was too nervous because he had lost all his money and two of his brothers in the earthquake. He was usually bad-tempered to me. I remember that he hit me and my mother for small excuses. I grew up in that difficult situation and its result was that I couldn't study at school. I always started fights at school. Now I am unemployed." (Code 5)*

**Internal Factors**

The internal factors affecting the life process of children who survived the earthquake are ‘understanding threats’, ‘fatalism’ and ‘dangerous nostalgias’.

**Understanding Threats**

For children earthquake survivors, facing stressful events, experiencing failures in family and social life, and emotional and educational breakdowns over the course of their lives cause them to feel threatened. The intensity of the feeling of threat shifts according to the context of each individual’s condition. Thus, the intensity and type of stressors influence the intensity of the feeling of being threatened. Feeling threatened affects these survivors’ performance in rebuilding their lives after the earthquake.

*"I feel so impatient. Once I was supposed to marry a girl but she left me, I experienced a feeling exactly like which I experienced at the time of earthquake. I reviewed all of my miseries. I felt lonely and threatened very much. I was depressed and devastated for some days." (Code 15)*

**Fatalism**

Believing in fate and luck are personal factors affecting the life process of these children. According to participants, feeling unlucky suppresses the opportunity to build personal, family and social dimensions of life. It can also cause depression, isolation and social isolation.

*"If I was lucky, why should I have been so miserable from childhood? Whatever I do, my bad luck and a part which has been already written in my destiny is with me." (Code 5)*

**Dangerous Nostalgias**

Internal factors which affect the life process of these survivors include breaks in time and retrieving memories of the earthquake when facing similar scenes or events.

*"One day, a motorbike had an accident with a car in the street. The motorbike rider's leg was stuck under the car's tire. As I saw this scene, I remembered my mum, her leg was stuck under the rubble of our house. The motorbike rider's shouts reminded me of stressful scenes and my mum's shouts. I felt so bad. I cried for many days." (Code 13)*

**External Factors**

‘Delayed relief activities’ and ‘non-specific care’ are external factors which affect the life process of children who survived the Manjil earthquake.

**Delayed Relief Activities**

A remarkable finding in the data is that often, treatment measures are delayed due to children's inability in expressing their immediate needs. This problem is worse in children with injured parents or for those who have lost their parents. Parents help aid groups in considering their children's needs with the knowledge they have about their psychological characteristics or physical problems. Delayed diagnosis or treatment of different problems of these survivors could have serious and persistent consequences for them.

*"I was there when they took a 7-year-old child to the hospital. The child cried and couldn't answer them when they asked questions. Nurses didn't even know what medicine the child was allergic to. I remember the doctor gave the child a medicine. The poor child was shocked immediately and died, and it was too painful for me. If they hadn't given the drug, the child wouldn't have been dead." (Code 10)*

**Non-Specific Care**

Health care is not often provided to alleviate the physical and mental needs of children who have survived an earthquake. This reduces the quantity and quality of services. Most attention is focused on mere physical needs, and the mental and emotional needs of these children are neglected. In the area of physical health care, aid workers forget some children, or at times offer them defective or delayed care due to their lack of knowledge of specific care for children.

*"They took me to the hospital. I heard two doctors saying that if the aid workers had paid more attention or been trained, they wouldn't have cut a woman's leg who was on a bed next to me." (Code 5)*

**Modifying Factors**

**‘Palliative self-care’ **and **‘Internal evolution’** are modifying factors in the life process of children who survived the earthquake.

**Palliative Self-Care**

The lack of long-term social and family services leads to self-action to have a safe and normal social and family life. Children seek different strategies to rebuild various aspects of their lives during different life stages. This category consists of three subcategories including **‘rescue efforts**’, **‘religious strategies’ **and **‘resilience’**.

**Rescue Effort**

The development of social communications, starting a family, having children and maintaining emotional ties are some of the strategies used by children in rebuilding their lives after earthquake. On the other hand, being optimistic in life, strengthening the ability of forgiveness, being involved in charity works, and public participation have calmed these people. According to participants, empathy is formed in peer groups because of their common pain. By developing social relationships, these people look for those who can be understood easily by them due to the common experience they share. Sharing memories of childhood and using coping strategies proposed in these groups improves their mental relaxation.

*"At the moment, I am a stepmother of three orphans. I don't have money, but this makes me feel good. My sister was six when she died, and I was 7 [at the time of earthquake]. I do it to make her happy." (Code 6)*

Some of the earthquake survivors who are capable of continuing their education attempt to help others by studying. Some even try to make their bereaved or dead parents happy.

*"I tried to be a doctor. Any patient who is visited by me to me and doesn't have money, I don't charge him. I cooperate with the Red Cross voluntarily. If my family had gotten treatments in time, they would have been alive right now. I became a doctor to help my people if necessary." (Code 7)*

**Religious Strategies**

One of the strategies for dealing with the persistent crisis of an earthquake is resorting to religious beliefs. Strengthening religious beliefs by reading prayer books and such religious texts as the Quran, participation in religious ceremonies and saying prayers are some of the religious strategies reportedly used by those who survived the earthquake in order to cope with multiple physical, mental and social problems. On the other hand, fatalism and 'submission to the will of God' help these children deal with problems after the earthquake.

*"As I grew up, I realised that God is the only refuge. Whenever I have problem I say prayers instead of crying and sadness. These beliefs help me out of all pains." (Code 1)*

**Resilience**

Resilience is one of the other strategies that children who survived the earthquake use in the process of rebuilding their lives. Resiliency is the result of a shift from being risk-oriented to coping with and modifying stress factors, which leads to the preservation and promotion of health in many aspects. Patience and perseverance, improving self-confidence, seeking familial and social support, and optional forgetfulness are some aspects of resiliency. These earthquake survivors often try to forget what happened in the past through emotional suppression.

*"Whenever I give up, I go to my friends and family. My mother understands me very well. She knows how much I was hurt then because I was a kid. I unburden myself [in] this way, and I have withstood many times [through] the help of her." (Code 8)*

**Internal Evolution**

Internal evolution refers to self-monitoring and positive psychological changes related to the earthquake. Self-monitoring and knowing oneself, others and God are some of the reported positive consequences of the earthquake. These respondents believe that knowing themselves and God gives them the power to deal with the chaos and emotional gaps caused by the earthquake.

*"The earthquake was a very bitter memory. But it built me. It made me resistant. It made me appreciate people. It made me more patient. It made me experience things which were productive. When I wonder, I think that it taught me that such moments won’t happen again. I knew my abilities and myself. These made me successful." (Code 7)*

## Discussion

According to the findings of this study, the first step in the lives of children who have survived the Manjil earthquake involves the unexpected encounter with the disaster. King (2006) believed immediately after the incident, the children experienced different reactions of shock and horror. These emotional reactions include a range of physiological, psychological, social and behavioural reactions^13^. More than other age groups, children are more prone to distress when faced with an earthquake disaster^14^. The results this study showed that in the early ravages caused by the earthquake, the loss of parents and shelter may have put the children at risk of harm and mistreatment. Therefore proper planning in crisis management, paying attention to the characteristics and needs of vulnerable groups including children, and comprehensive support can reduce the risk of mistreatment^15^. Based on the results of this study, there is a need for plans to provide long-term services.

It is important to note that threatening factors are persistent due to the long-term and sometimes permanent effects of earthquake disasters, and children are in great need of care at all stages of their lives. Unfortunately, rehabilitation and long-term services are marginal in disaster management planning^16^. Under these circumstances, there is a significant increase in mortality and a notable decrease in the physical, psychological and social function of these children^17^. Even if these services exist in society, they do not cover all survivors due to their unavailability and young age. Therefore, providing and sustaining long-term rehabilitation services must be considered as priorities when it comes to children survivors of natural disasters^18^.

The results this study also indicated that temporary social support and the subsided emotions of people and charities over time cause survivors to be deprived of support services, especially long-term emotional support. According to this group of survivors, after almost 24 years, the disaster has been forgotten and has disappeared from people's minds. Bolton et al ( 2000) believed this is associated with the lack of understanding by people, especially non-peers^19^. Concealing the feelings of others and social isolation are some of the strategies that this group of survivors may use to face such reactions^20^.

Further, this study's results showed that children whose other relatives are given custody and children who are adopted have the most problems. Since relatives are given priority in adopting a child, in most cases the economic status, emotional situation and social health of these families are not considered before the adoption. The new family members cannot provide a safe haven for these children because they are in crisis and are suffering from many financial, physical and mental problems. These children may experience violence, misbehaviour and secondary displacement in new families^21^. In such circumstances, these children feel that they are a burden, and experience a lack of opportunity in education, marriage, or suitable occupations^22^.

Regarding the fact that many of the participants of the present study have lost one or both of their parents in the earthquake, and given that some live with family members in an environment which is not physically, mentally and emotionally stable, most of these survivors face severe emotional issues as their future relationships are threatened. The results of McDermott's (2005) study showed that distrust of friendly and effective communication with others on one hand and emotional gaps on the other can all cause behavioural and emotional conflicts, and can bring about short-term emotional and social communication issues^23^.

Failures in communication and constructive attachments cause these child survivors to experience failure in marriage and family life^24^. Therefore, comprehensive services such as counselling, health care and welfare services are necessary^25^. This study's findings also showed that sometimes, parents do not succeed in their parental roles in supporting their children due to the distress they experience as a result of the earthquake. In such cases, these parents in turn can pose a risk to their own children. The results of Gertrud's (2004) study showed that parents who were themselves severely impacted by disaster reported a reduced ability to assess their children's reactions, and thereby were unable to provide optimal care^2^. Interestingly, these parents’ support strategies mirrored the early intervention recommendations put forward in the psychological first-aid guidelines which is a well-accepted and promising practice for helping children after disasters^2^. In the present study, mental nostalgia is one of the internal factors affecting the lives of those who survived the earthquake as children. These people retrieve memories when they face scenes that they consider as similar to events at the time of the earthquake. Hafstad et al (2004) believed nostalgia of past memories threatens their mental and emotional health during different stages of their lives^2^.

Believing in bad luck is another internal factor affecting the lives of those who were children earthquake survivors. Similar studies have shown that these beliefs can suppress a person's effort in coping with stressful atmospheres, and can keep individuals from trying to improve their family and social lives^22^. Believing in bad luck can also deter this group of people from pursuing their previous dreams and wishes, as well as from social communication^26^. As the data in this study showed, one of the factors causing threats to the health of these children survivors is non-specific care. It should be noted that ignoring the needs of these survivors, considering the services superficially and providing those services temporarily may threaten their health in various aspects^27^.

In order to prevent more permanent damages to child survivors, we have to train individuals, aid workers and volunteers to bring up people from under the rubble correctly, identify specific needs of children survivors, and further care provisions^28^. Participants in this study have tried to resort to strategies such as the development of social communication, spirituality, and psychological strengthening to maintain stability and balance in their individual, family and social lives. In fact, other studies have emphasised religious beliefs as a facilitator of these survivors’ life processes^29^. Developing social relationships and friendships help improve the quality of survivors’ lives^30^.

## Conclusion

According to the results of the present study, providing non-specific and temporary services is one of the factors affecting the life processes of children who have survived the Manjil earthquake. Training aid workers to consider the specific needs of children at the time of rescue operations could contribute to improving the health of this group in different aspects. Considering effective and comprehensive rehabilitation programs in Disaster Management policymaking can prevent persistent complications caused by earthquakes and other natural disasters. The results of this study also showed that some of the factors threatening the health of children earthquake survivors include homelessness, living in care centres, and living with risky parents. Planning and taking action to identify misbehaviour in this group of children, as well as raising public awareness (particularly for parents) on how to manage a disaster, are public health priorities. Providing public counselling services to children survivors as well as their parents will help to solve the potential psychological problems that threaten the well-being of children survivors.

Further, this study showed that children have tried to use different strategies in the process of their lives to maintain and promote their personal and social life. Strengthening religious beliefs and encouraging children survivors to participate in social groups (especially peer groups) will help them to feel understood, safe and alleviated of many negative feelings resulting from their experience of a natural disaster.

## Appendix A: Interview Guide Questions

1- Can you tell me about your experiences after the earthquake?

2- What happened to you at the moment the earthquake happened?

3- Please talk about the harms happened to you.

4- How do you describe the life after the earthquake?

5- What has helped you to deal with the earthquake?

6- What does earthquake mean to you?

7- How was your life?

8- Would you please review a day of your life?

9- Which factors have helped you at the moment of the earthquake or after that?

## Limitation

Among the limitations of this study, restrictions in generalization of the results can be mentioned. As interview method was used for data collection, the veracity of the interviewees might have affected the results. Therefore, conducting more studies focusing on triangulation methods is suggested.

## Corresponding Author

Dr Fatemeh Ghaffari. Email: ghafarifateme@yahoo.com

## Data Availability

All relevant data are within the paper.

## Competing Interests

The authors have declared that no competing interests exist.
